# Anesthesia outcomes of pregnant women with spinal diseases: a single-center case-series study

**DOI:** 10.1186/s40981-023-00648-2

**Published:** 2023-08-30

**Authors:** Adila Yakhup, Hisako Okada, Izumi Kawagoe, Hiroyuki Sumikura

**Affiliations:** 1 Department of Anesthesiology and Pain Medicine, Juntendo University Graduate School of Medicine, 2-1-1 Hongo, Bunkyo-Ku, Tokyo, 113-8431 Japan; 2 Department of Anesthesiology and Pain Medicine, Juntendo University Nerima Hospital, 3-1-10 Takanodai, Nerima-Ku, Tokyo, 177-8521 Japan; 3 Department of Anesthesiology and Pain Medicine, Juntendo University Urayasu Hospital, 2-1-1, Tomioka, Urayasu, Chiba, 279-0021 Japan; 4 Department of Anesthesiology and Pain Medicine, Juntendo University Hospital, 3-1-3 Hongo, Bunkyo-Ku, Tokyo, 113-8431 Japan

**Keywords:** Neuraxial anesthesia, Labor analgesia, Pregnancy, Spinal disease, Scoliosis

## Abstract

**Background:**

Neuraxial anesthesia is widely used as the most effective and standard method in obstetric anesthesia. However, there is a concern that neuraxial anesthesia may be technically difficult or ineffective in pregnant women with spinal disease. Therefore, this study aimed to investigate the implementation rate of neuraxial anesthesia among pregnant women with spinal diseases and their success rate at our institution.

**Methods:**

The subjects of this study were pregnant patients who delivered at Juntendo University Nerima Hospital between April 2017 and December 2020. After obtaining ethics committee approval, data were collected from patients’ medical records.

**Results:**

Of the 2682 pregnant women who delivered, 1550 underwent preanesthetic evaluation. There were 42 deliveries in 39 pregnant women with spinal diseases (1.7% of all pregnant women and 2.7% of those who underwent preanesthetic evaluation). The diagnoses included adolescent idiopathic scoliosis (51.3%), lumbar disc herniation (23.1%), and others. The mode of delivery was the elective cesarean section in 5 cases, emergent cesarean section in 8 cases, and vaginal delivery in 29 cases. Only one case required general anesthesia. Of the 38 cases of labor analgesia, the neuraxial block was inadequate in 3 cases (7.9%) and technically difficult in 3 cases (7.9%). However, the patients complained of no lower extremity neuropathy, infection, or inadvertent dural puncture.

**Discussion:**

Neuraxial anesthesia was an option in most cases, even in pregnant women complicated with spinal disease, if an anesthesiologist’s plan before delivery after careful preanesthetic evaluation.

## Background

Neuraxial anesthesia is the most effective and standard method in obstetric anesthesia, including anesthetic management for cesarean section and pain management during delivery [1–3]. For pregnant women with some condition affecting the spine or spinal cord, however, neuraxial anesthesia is regarded as being technically difficult or ineffective [4–7]. Many anesthesiologists believe that neuraxial anesthesia should be avoided and are less likely to provide it for pregnant women with spinal diseases, even on request for labor analgesia during delivery [8–10]. Anesthesiologists choose general anesthesia over neuraxial anesthesia when a cesarean section is required for those women [11].

More recently, however, the importance of neuraxial anesthesia in obstetric anesthesia has been recognized, and the widespread use of magnetic resonance imaging (MRI) as a diagnostic imaging method during pregnancy has made it possible to perform spinal imaging without exposing the mother or fetus to radiation. Therefore, the rate of the parturient receiving neuraxial anesthesia and its success rate among these patients may have changed compared to the past reports.

This study aimed to investigate the implementation rate of neuraxial anesthesia at delivery among pregnant women with spinal diseases and their success rate retrospectively at our institution.

## Methods

The subjects of this study were pregnant women who gave birth at Juntendo University Nerima Hospital between April 2017 and December 2020. After obtaining ethics committee approval (IRB no. E21-0161-N01), data were collected from the medical records.

First, anesthesiologists selected patients who underwent a prenatal evaluation before delivery. These patients included those scheduled for cesarean section, those who requested labor analgesia, and those with some risk (including spinal disease). These patients should visit the perinatal anesthesia outpatient clinic by 36-week gestation. However, even if the patient had not visited the outpatient perinatal anesthesia clinic by the time of admission for delivery, an anesthesiologist performed a prenatal evaluation in the ward after admission when he or she deemed it necessary. Among those patients, the presence or absence of a history of spinal disease was ascertained from the medical records of eligible patients, and patients with a history of spinal disease were selected. In addition, individual information on obstetric management, anesthesia management, and the outcome of spinal disease was collected from the medical records of these patients.

The epidural labor analgesia dosing regimen is as follows: an epidural catheter was placed in the L3/4 or L4/5 intervertebral space at the patient’s request. After a negative aspiration test, half doses of 10 mL of ropivacaine 0.1% containing 50 µg of fentanyl were administered at 5-min intervals to ensure no signs of local analgesic toxicity or intrathecal injection. When combined spinal-epidural analgesia is selected, 2.5 mg hyperbaric bupivacaine, 10 µg fentanyl, and 1.3 mL saline are administered intrathecally. Programmed intermittent epidural bolus (PIEB) was then initiated in women whose pain scores decreased within 20 min after the end of the loading dose. The programmed bolus dose was fixed at 5 mL of ropivacaine 0.085% containing 2 μg/mL of fentanyl. The first bolus was delivered 1 h after the loading dose. The PIEB interval was set at 60 min. The delivery system was programmed to inject 5-mL PCEA boluses of the same solution with a lockout interval of 15 min and to deliver a maximum volume of 20 mL per hour.

Effective analgesia was defined as no additional bolus or administration of up to two manual boluses after the start of patient-controlled epidural analgesia until the patient demonstrated complete cervical dilation. For breakthrough pain, either 10 mL of an additional bolus, 5 to 10 mL of 0.2% ropivacaine, 25 to 50 µg of fentanyl, 5 mL of 1% xylocaine, or a combination was used. Pain scores, sensory block levels to alcohol swabs, motor block degree, blood pressure, and maternal and fetal heart rate were assessed every 1 or 2 h.

## Results

Of the 2682 pregnant women who gave birth during the study period, 1550 patients underwent a prenatal evaluation by anesthesiologists. Of whom, 1537 received it before admission for scheduled cesarean Sect. (703 cases) and others (834 cases) and 13 after admission. Of the 1550 patients, 42 (2.7%) were identified as having a history of spinal disease; three had given birth twice, so the number of patients with spinal disease was 39. The diagnoses of spinal disease included adolescent idiopathic scoliosis (AIS) in 20 patients (51.3%), lumbar disc herniation (LDH) in 9 patients (23.1%), and others in 10 patients (Table 1).

**Table 1:** Details of spinal diseases and surgical history

Diagnoses	Frequency (total/%)	Number of patients with surgical history	Surgery location on the spine
Adolescent idiopathic scoliosis	20 (51.3)	3	Th3-L2, Th3-L1, Th4-L4
Lumbar disc herniation	9 (23.1)	0	-
Sciatica neuralgia	2 (5.1)	0	-
Spina bifida	2 (5.1)	2	Th12-L1, Th4–6
Tethered cord syndrome	1 (2.5)	0	-
Lumbar compression fracture	1 (2.5)	0	-
Cervical spondylosis	1 (2.5)	0	-
Cervical disc herniation	1 (2.5)	0	-
Spontaneous spinal epidural hematoma	1 (2.5)	1	C5–6
Cavernous hemangioma	1 (2.5)	1	C2

The percentages in parentheses indicate the proportion of each diagnosis among the 39 patients included in the study

Of the 39 pregnant women confirmed to have spinal disease before delivery, seven had undergone spinal surgery. Thirteen of the 39 pregnant women with confirmed spinal disease had consultations with other departments (11 orthopedic surgeons and two neurosurgeons) before delivery. Thirteen pregnant women underwent additional imaging studies before delivery, including MRI (5 women) and X-ray (7 women). In addition, spinal cord angiography was performed on one woman with a history of recurrent spontaneous cervical epidural hematoma between her first and second pregnancies.

The final mode of delivery was elective cesarean section, emergent cesarean section, and vaginal delivery in 5, 8, and 29 cases, respectively (Table 2). General anesthesia was selected only in 1 case with a history of recurrent spontaneous spinal epidural hematoma [12].

**Table 2:** The mode of delivery and anesthesia methods

**Type of anesthesia**	**Mode of delivery**					
	**Elective cesarean section**	**Emergent cesarean section**		**Vaginal delivery**		**Total**
		**After labor analgesia**	**Without labor analgesia**	**After labor analgesia**	**Without labor analgesia**	
General	1	0	0	0	0	1
Epidural	0	5	0	22	0	27
Spinal	3	0	3	0	0	6
CSEA	1	0	0	4	0	5
None	0	0	0	0	3	3
Total	5	5	3	26	3	42

*CSEA* combined spinal-epidural analgesia

### Cesarean section (Table 3)

**Table 3:** Indications for cesarean section and anesthetic considerations by diagnosis

Subject number	Diagnoses	Spinal level	Lumbar Cobb angle	Elective/emergent	Indication for CS	Type of anesthesia	Reason for type of anesthesia
1	AIS (postoperative)	Th4-L4	< 10	Elective	Previous CS	Spinal	Spinal instrumentation, but intact L4/L5 intervertebral space
2	AIS	Th7-L3	10	Elective	Post myomectomy	Spinal	Well palpable spinous process
3	AIS	Th11-L4	< 10	Emergent	CPD	Epidural	Labor analgesia
4	AIS	Lumbar	< 10	Emergent	Arrest of labor	Epidural	Labor analgesia
5	AIS	Th12-L4	< 10	Emergent	NRFS, category 1 emergent CS	Epidural	Labor analgesia
6	AIS	Th2-L4	< 10	Emergent	HDP	Spinal	Well palpable spinous process
7	AIS	Th2-L5	< 10	Emergent	NRFS	Spinal	Well palpable spinous process
8	LDH	Lumbar	-	Emergent	Arrest of labor	Epidural	Labor analgesia
9	LDH	Lumbar	-	Emergent	CPD	Epidural	Labor analgesia
10	SSEH	C5–6	-	Elective	Avoid pushing for recurrence of epidural hematoma	General	Avoid rebleeding in spinal canal
11	Lumbar compression fracture	Th11-L3	-	Elective	Previous CS	Spinal	Osteogenesis imperfecta but well-palpable spinous process
12	Cavernous hemangioma	C2	-	Elective	Previous CS	CSEA	Two previous cesarean deliveries with CSEA
13	Cervical disc herniation	Cervical	-	Emergent	CPD	Spinal	Well palpable spinous process

*AIS* adolescent idiopathic scoliosis, *LDH* lumbar disc herniation, *SSEH* spontaneous spinal epidural hematoma, *CPD* cephalopelvic disproportion, *NRFS* nonreassuring fetal status, *HDP* hypertensive disorders of pregnancy, *CS* cesarean section

Of the eight patients who had emergent cesarean sections, epidural labor analgesia had been introduced in five of them. In all of them, the cesarean section was successfully managed using the indwelling epidural catheter. In the three cases where epidural labor analgesia had not been introduced, they were successfully anesthetized for cesarean section with single-shot spinal anesthesia.

### Vaginal delivery (Table 4)

**Table 4:** Management of anesthesia for vaginal delivery in challenging cases

Case	Age	History of pregnancy (para)	Clinical diagnoses	Neurological findings and symptoms	Planned anesthesia	Switch to alternative plan	Consequence
1	34	0	AIS, obesity BMI (35.7)	Difficult palpitation. T7–T11 thoracic Cobb angle 43°. T11-L4 thoracolumbar Cobb angle 41°	EDB	NA	EDB at L3/4 after multiple attempts
2	33	0	LDH	Narrow intrathecal space at L4/5. Pain in the back of the right thigh and calf	DPE	EDB	CSF not obtained w/DPE. Sufficient analgesia w/EDB
3	36	1	Spina bifida	Postoperative change at Th12-L1. Intact epidural space below L2. Sensory loss below Th12/L1, with preserved deep sensation but impaired temperature and pain perception	CSEA below L2/3	CSEA at L4/5	Difficult EDB at L3/4. Sufficient analgesia w/CSEA
4	32	0	AIS	Postoperative instrumentation at Th3-L2	CSEA at L3/4	EBD at L3/4	No cold loss above Th12. But patient satisfied with receiving labor analgesia
5	40	0	LDH	Narrow intrathecal space at L5/S1. Previous numbness and pain at right big toe. Pain in the lower right back	EDB at L3/4	Redo CSEA at L4/5	Poor spread below L5 with EDB. Sufficient analgesia after CSEA
6	32	0	Tethered cord syndrome	Conus medullaris terminates at inferior border of L4 vertebral body. Tarlov cyst at sacral area. Low back pain	EDB at L2/3	EDB replacement. Additional pudendal nerve block	Severe pain during the second stage of labor. Not fully relieved by alternative blocks

*EDB* epidural block, *DPE* dural puncture epidural, *BMI* body mass index, *MRI* magnetic resonance imaging, *CSF* cerebrospinal fluid, *NA* not available, *w/* with

**Fig. 1:**
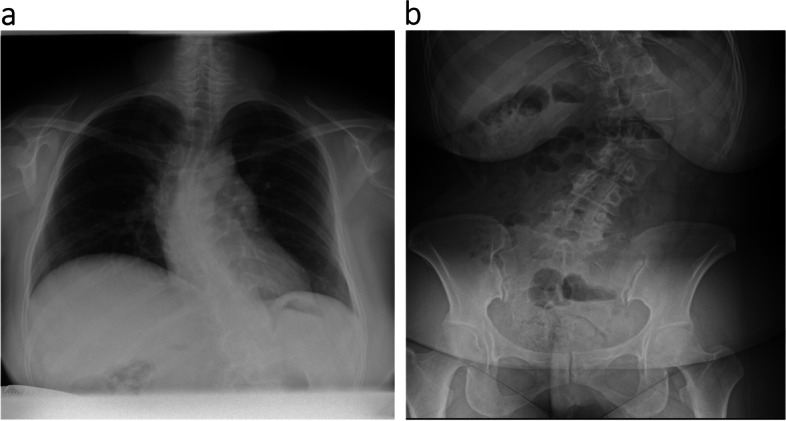
X-ray exams for a pregnant woman with moderate scoliosis. **a** Chest X-ray before delivery revealed that her thoracic Cobb angle was 43°, and her upper lumbar spine was confirmed to have scoliotic change. **b** Abdominal X-ray after delivery revealed that her lumbar Cobb angle was 41° and rotated

**Fig. 2:**
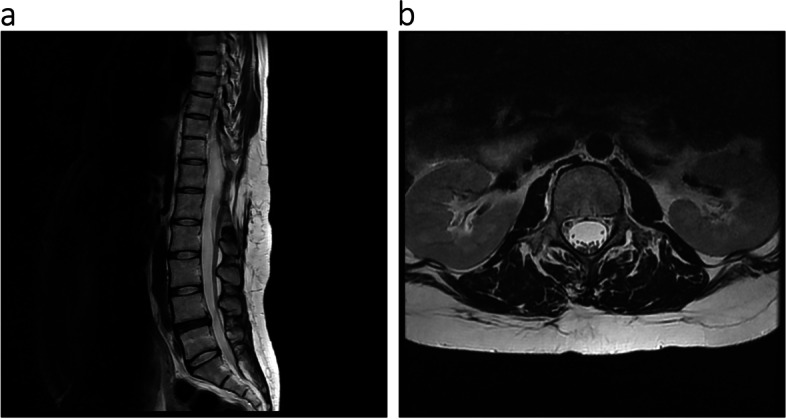
Magnetic resonance imaging for pregnant woman with a history of spina bifida. **a** Sagittal MRI imaging revealed postoperative change at T12/L1 intervertebral space. Epidural space was not apparent from T11 to L2 but became apparent below L2. **b** Both anesthesiologists and orthopedic surgeons confirmed the L3/4 intervertebral epidural space

Epidural labor analgesia was introduced in 26 cases among the 29 patients who delivered vaginally. Epidural catheter placement was successful in all patients, with no complications such as dural puncture, neuropathy, or infection; however, epidural puncture was somewhat difficult but provided adequate analgesia in 3 (cases 1–3) and insufficient analgesia in 3 (cases 4–6) (Table 4). In brief, multiple punctures from multiple intervertebral spaces were required in the case of AIS (case 1, Fig. 1a,b). The initial plan of dural puncture epidural anesthesia was changed into epidural anesthesia in the case of LDH because of paresthesia and a lack of backflow of the cerebrospinal fluid during dural puncture after confirming the epidural space (case 2). Combined spinal-epidural analgesia (CSEA) below L2/3 intervertebral space was planned in a case of spina bifida after confirming an intact epidural space from L2 to the caudal region. CSEA was performed at L4/5 after multiple punctures from multiple intervertebral spaces (case 3, Fig. 2a, b).

**Fig. 3:**
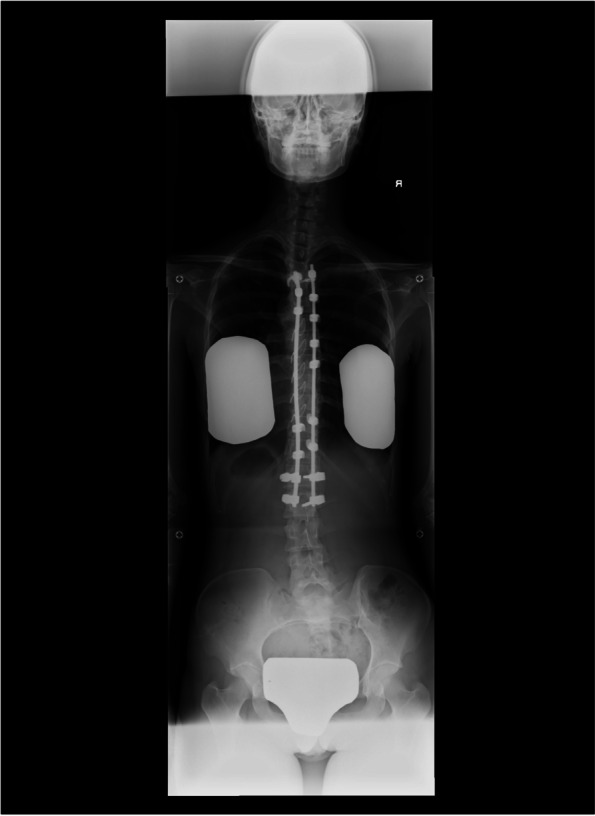
Spinal X-ray for a pregnant woman after fixation surgery. Spinal X-ray of surgical adolescent idiopathic scoliosis demonstrates instrumentation from Th3 to L2

**Fig. 4:**
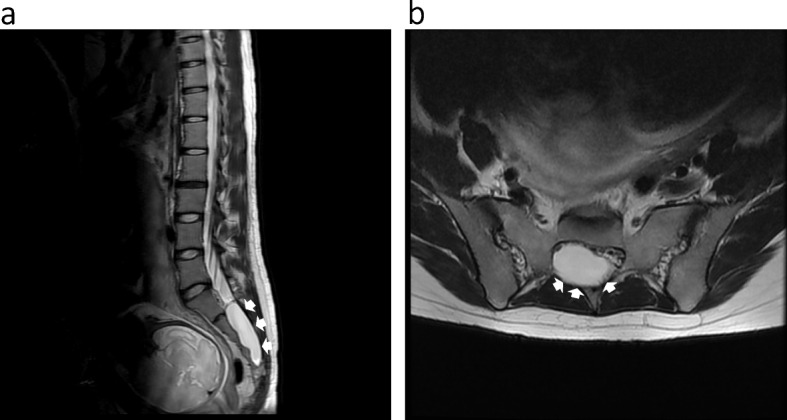
Magnetic resonance imaging for a pregnant woman with tethered cord syndrome and Tarlov cyst. **a** Sagittal T2-weighted image demonstrates fixation (tethering) of filum terminale on the anterior spinal canal, and the conus medullaris ended at L4. **b** Coronal image demonstrates the Tarlov cyst (sacral cyst in the spinal canal, arrows)

Loss of cold sensation did not extend above Th12 after epidural catheterization from L3/4 in a patient with a history of spinal fusion surgery for AIS. She complained of insufficient pain relief, especially during the first stage of labor, although she was satisfied with good analgesia in the second stage of labor (case 4, Fig. 3). Although cold hypoesthesia was achieved to Th9 after epidural catheterization at L3/4, pain control was ineffective on the lower side below L5 in a patient with LDH. CSEA at L4/5 provided satisfactory pain relief (case 5). An epidural catheter was inserted at L2/3 after the onset of labor in a patient with tethered cord syndrome, which provided sufficient pain control in the first stage of labor. However, she complained of severe pain in the second stage of labor, even after adding the pudendal nerve block (case 6, Fig. 4a, b).

## Discussion

This retrospective study showed that of the 1550 patients who underwent prenatal anesthesia evaluation, 42 cases (2.7% or 1.7% of all pregnant women during the study period) had a history of spinal disease. General anesthesia for cesarean section was performed in only 1 of 13 cases. Neuraxial labor analgesia was performed in 38, with six challenging cases (15.8%) being difficult punctures or inadequate analgesia but no serious complications. This result indicates that neuraxial anesthesia can be selected for delivery in many pregnant women with a history of spinal disease if anesthesiologists conduct a thorough preoperative evaluation and develop an anesthetic plan. These results are similar to those by Villevieille et al., which indicated technical difficulties (9%) and insufficient efficacy (9%) in these patients and argued that epidural labor analgesia is not contraindicated, as no serious complications were observed [7].

Although the importance of neuraxial anesthesia in obstetric anesthesia is widely recognized, the indication for neuraxial anesthesia should be carefully considered in pregnant women with spinal disease.

The most frequent spinal disease in this study was AIS (20 patients), and it was revealed that 3 of these patients had undergone surgery for AIS. For these patients, the individual anesthetic plan was made for the case of cesarean section or labor analgesia after accurate identification of the surgical site, and consent was obtained regarding the indication of neuraxial anesthesia. It is worth noting that surgical techniques have evolved, and there are now situations where epidural anesthesia may be applicable even after AIS surgery [13]; therefore, evaluation with an orthopedician is needed. During the prepartum anesthetic evaluation, it was determined that 11 patients had a mild degree of AIS. They were also informed of their higher risk for a difficult procedure or insufficient analgesic effect compared to patients without AIS. As a result, 17 of the 20 patients underwent neuraxial anesthesia at delivery. However, only one patient had difficulty with the puncture, and one patient had poor pain control, with no complaints from any patients.

Similar to the present results, Bauchat et al. reported that induction of neuraxial anesthesia for labor analgesia was more difficult in pregnant women who had undergone spinal fusion for AIS than in normal pregnant women without AIS or other preexisting conditions, with difficulty in pain control which occurred in about 12% of cases [14]. However, both their report and ours indicate that neuraxial anesthesia can be selected in many cases if the anesthetic plan is discussed with the patient beforehand, and additional examinations are conducted according to the severity of the AIS.

LDH (9 patients) was the second most frequent spinal disease among pregnant women. Since the epidural block is the treatment for LDH, there should be no issue in choosing neuraxial anesthesia at delivery if prior evaluation and explanation to the patient are provided. Two other patients had sciatica neuralgia but received a neuraxial procedure at delivery, as an epidural block is also the preferred treatment for sciatica neuralgia.

Although spina bifida (2 patients) is relatively rare, special care should be taken when performing neuraxial anesthesia. A review by Sivarajah et al. noted that epidural anesthesia in patients with spina bifida occulta is often uneventful. However, spinal cord injury is possible without MRI imaging confirmation if tethered cord syndrome is present [15]. Indeed, spinal cord injuries due to neuraxial anesthesia in pregnant women with undiagnosed latent spina bifida and tethered cord syndrome have been reported [16, 17]. Therefore, in pregnant women suspected of having spina bifida, confirmation of the location of the conus medullaris by MRI is essential to prevent nerve injury, and analgesic methods other than neuraxial anesthesia should be considered if the risk of spinal cord injury is high. It has also been reported that the frequency of postpartum back pain is increased in pregnant women after spinal fusion surgery [18, 19], and patients may need to be informed before delivery about the possibility of back pain unrelated to anesthesia.

For the patient with a rare history of spontaneous spinal epidural hematoma during the cesarean section, the anesthesiologists, orthopedics, and obstetricians chose general anesthesia. This choice would be a good example of how neuraxial anesthesia could have been avoided in a risky situation with proper prepartum evaluation by an anesthesiologist.

In 2016, our hospital began offering 24-h labor analgesia service. As part of this program, it began offering prenatal anesthetic evaluations in the obstetric anesthesia outpatient clinic for pregnant women with comorbidities and patients who wish to have labor analgesia. As a result, patients with spinal disease could be identified before delivery, and spinal disease was recognized immediately before emergency cesarean section, reducing the difficulty in handling such patients. In addition, for pregnant women who wished to have labor analgesia, neuraxial anesthesia could be selected flexibly on a case-by-case basis by explaining the risks and benefits of neuraxial anesthesia after prior evaluation of spinal disease. In the future, intravenous patient-controlled analgesia (IV-PCA) or nitrous oxide should be offered for cases where neuraxial labor analgesia is contraindicated. However, at this point, we have not offered these options at our facility because we cannot adequately guarantee their safety.

In recent years, the use of ultrasound guidance in neuraxial anesthesia has become increasingly prevalent. Pre-puncture ultrasound examinations can provide crucial information such as the identification of the midline of the spine, precise intervertebral space, prediction of the depth of the epidural space, and determination of the needle insertion angle. This has established ultrasound guidance as a key tool for delivering high-quality healthcare [20, 21]. Particularly in cases where patients are obese or palpation for landmarks is difficult, the use of ultrasound-guided punctures can reduce the risk of vascular puncture and decrease the incidence of postpartum back pain and headaches, demonstrating its significant advantages in enhancing safety [22]. However, the application of ultrasound-guided neuraxial puncture has yet to become as widespread and used as the landmark technique due to the lack of adequate training for every anesthesiologist and the inability to make the necessary preparations as soon as they detect a puncture difficulty.

**Fig. 5:**
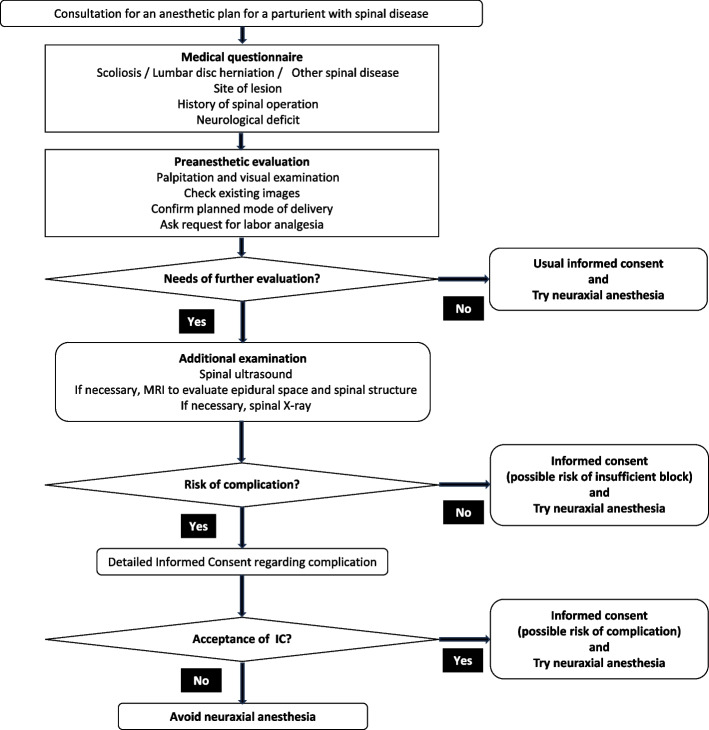
Tentative flowchart of decision-making for neuraxial analgesia in parturients with spinal diseases. Based on this flowchart chart, we need to make the final decision on the anesthesia method after comprehensively assessing the patient’s intention, the medical indications for neuraxial anesthesia, and the skill of the anesthesiologist in charge of the patient

Through this study, we have developed a tentative flowchart for performing neuraxial anesthesia in pregnant women with spinal disease (Fig. 5). Based on this flowchart chart, we need to make the final decision on the anesthesia method after comprehensively assessing the patient’s intention, the medical indications for neuraxial anesthesia, and the skill of the anesthesiologist in charge of the patient. Further research is needed to make this flowchart complete.

The following caution should be exercised when interpreting the results of this study. First, the rate of spinal disease in this study may have been reported as higher than the standard rate. Originally, our institution is affiliated with a university hospital with a high proportion of pregnant women with complications and a low proportion of healthy pregnant women with low risk. It is worth noting that labor analgesia is not widely available in Japan, which may have resulted in a higher concentration of pregnant women with spinal complications seeking care at our facility. Second, the safety of neuraxial anesthesia may have been overemphasized. Although we provided neuraxial anesthesia to pregnant women with spinal disease with the utmost care, we cannot guarantee its safety based on our results because of the small number of cases. Therefore, the choice of general or neuraxial anesthesia for cesarean section should be determined case by case. For pregnant women who wish to have labor analgesia, IV-PCA or nitrous oxide should also be offered as an option.

This study suggests that appropriate anesthetic evaluation before delivery can contribute to the safety and comfort of delivery for at-risk pregnant women. Although this study focused on spinal disease, the same should be true for other at-risk pregnant women. Labor analgesia provides a good chance to assess prenatal anesthesia in pregnant women. Labor analgesia is hoped to become more widespread in Japan, and anesthesiologists will routinely perform prenatal evaluations on all pregnant women.

## Data Availability

The datasets used and analyzed during the current study are available from the corresponding author upon reasonable request.

## Abbreviations

MRI: Magnetic resonance imaging
LDH: Lumbar disc herniation
CSEA: Combined spinal-epidural analgesia
SSS: Single-shot spinal anesthesia
BMI: Body mass index
DPE: Dural puncture epidural anesthesia
CSF: Cerebrospinal fluid
IV-PCA: Intravenous patient-controlled analgesia
CPD: Cephalopelvic disproportion
NRFS: Non-reassuring fetal status
HDP: Hypertensive disorders of pregnancy
EDB: Epidural block
CS: Cesarean section
